# Correction: Qiu et al. Genistein Modified with 8-Prenyl Group Suppresses Osteoclast Activity Directly via Its Prototype but Not Metabolite by Gut Microbiota. *Molecules* 2022, *27*, 7811

**DOI:** 10.3390/molecules29245818

**Published:** 2024-12-10

**Authors:** Zuo-Cheng Qiu, Feng-Xiang Zhang, Xue-Ling Hu, Yang-Yang Zhang, Zi-Ling Tang, Jie Zhang, Li Yang, Man-Sau Wong, Jia-Xu Chen, Hui-Hui Xiao

**Affiliations:** 1 Guangzhou Key Laboratory of Formula-Pattern of Traditional Chinese Medicine, School of Traditional Chinese Medicine, Jinan University, Guangzhou 510632, China; zcqiu@jnu.edu.cn (Z.-C.Q.);; 2 Guangdong Provincial Key Laboratory of Traditional Chinese Medicine Informatization, Jinan University, Guangzhou 510632, China; 3 State Key Laboratory for Chemistry and Molecular Engineering of Medicinal Resources, School of Chemistry and Pharmacy, Guangxi Normal University, Guilin 541004, China; 4 College of Pharmacy, Jinan University, Guangzhou 510632, China; 5 State Key Laboratory of Chinese Medicine and Molecular Pharmacology (Incubation), Shenzhen Research Institute of the Hong Kong Polytechnic University, Shenzhen 518057, China; 6 Research Center for Chinese Medicine Innovation, The Hong Kong Polytechnic University, Hong Kong 999077, China

## Error in Figure

In the original publication [1], there was a mistake in Figure 5A as published. The pictures of 8PG at 5 × 10^−6^ M and G 10^−5^ M in Figure 5A are duplicated due to the wrong copying and pasting of pictures when organizing them into one figure using Adobe Illustrator software. The corrected Figure 5A appears below.

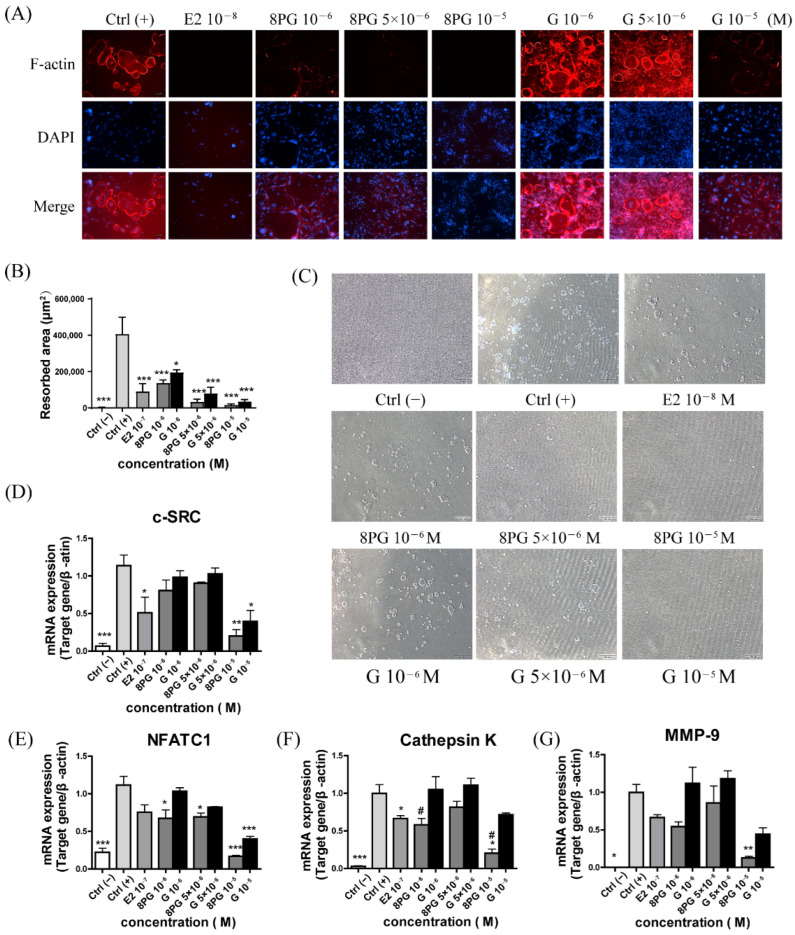



The authors state that the scientific conclusions are unaffected. This correction was approved by the Academic Editor. The original publication has also been updated.

## References

[1] Qiu Z.-C., Zhang F.-X., Hu X.-L., Zhang Y.-Y., Tang Z.-L., Zhang J., Yang L., Wong M.-S., Chen J.-X., Xiao H.-H. (2022). Genistein Modified with 8-Prenyl Group Suppresses Osteoclast Activity Directly via Its Prototype but Not Metabolite by Gut Microbiota. Molecules.

