# Supplementary material for: Genistein Modified with 8-Prenyl Group Suppresses Osteoclast Activity Directly via Its Prototype but Not Metabolite by Gut Microbiota
Source: Molecules. 2022 Nov 13;27(22):7811. doi: 10.3390/molecules27227811 (PMC9694937; doi:10.3390/molecules27227811)
Supplement: Supplementary file 1 [file molecules-27-07811-s001.zip › molecules-1951372-supplementary.pdf]

Supplementary Table

Table S1. Sequence of primers used in quantitative RT-PCR

Gene	Forward primers (5'-3')	Reverse primers (5'-3')
m-c-Src	CTTCGGAGAGGTGTGGATG	GTGCCTGGGTTCAGAGTTTT
m-NF-ATc1	TCCAAAGTCATTTTCGTGGA	CTTTGCTTCCATCTCCCAGA
m-MMP-9	CTGGACAGCCAGACACTAAAG	CTCGCGGCAAGTCTTCAGAG
m-Cathepsin K	GAAGAAGACTCACCAGAAGCAG	TCCAGGTTGGGCAGAGATT
m-GAPDH	CCGCAATAGACAAGGACAT	CTCGCAGAAGGTGAACTC
